# Genome-wide expression analysis reveals different heat shock responses in indigenous (*Bos indicus*) and crossbred (*Bos indicus* X *Bos taurus*) cattle

**DOI:** 10.1186/s41021-023-00271-8

**Published:** 2023-05-01

**Authors:** Basavaraj Sajjanar, Mohd Tanzeel Aalam, Owais Khan, Gunturu Narasimha Tanuj, Aditya Prasad Sahoo, Gundallahalli B. Manjunathareddy, Ravi Kumar Gandham, Sujoy K. Dhara, Praveen K. Gupta, Bishnu Prasad Mishra, Triveni Dutt, Gyanendra Singh

**Affiliations:** 1 Veterinary Biotechnology Division, ICAR-Indian Veterinary Research Institute, Izatnagar, Bareilly, 243122 Uttar Pradesh India; 2 ICAR- Directorate of Foot and Mouth Disease, Bhubaneswar, 752050 Odisha India; 3 ICAR-National Institute of Veterinary Epidemiology and Disease Informatics, Yelahanka, 560064 Karnataka India; 4 ICAR-National Bureau of Animal Genetic Resources, Karnal, 132001 Haryana India; 5 Physiology and Climatology Division, ICAR-Indian Veterinary Research Institute, Izatnagar, Bareilly, 243122 Uttar Pradesh India

**Keywords:** Heat stress, Dairy cattle, Immune response, Gene expression

## Abstract

Environmental heat stress in dairy cattle leads to poor health, reduced milk production and decreased reproductive efficiency. Multiple genes interact and coordinate the response to overcome the impact of heat stress. The present study identified heat shock regulated genes in the peripheral blood mononuclear cells (PBMC). Genome-wide expression patterns for cellular stress response were compared between two genetically distinct groups of cattle viz., Hariana (*B. indicus*) and Vrindavani (*B. indicus X B. taurus*). In addition to major heat shock response genes, oxidative stress and immune response genes were also found to be affected by heat stress. Heat shock proteins such as *HSPH1*, *HSPB8*, *FKB4*, *DNAJ4 and SERPINH1 *were up-regulated at higher fold change in Vrindavani compared to Hariana cattle. The oxidative stress response genes (*HMOX1*, *BNIP3*, *RHOB* and *VEGFA*) and immune response genes (*FSOB*, *GADD45B* and *JUN*) were up-regulated in Vrindavani whereas the same were down-regulated in Hariana cattle. The enrichment analysis of dysregulated genes revealed the biological functions and signaling pathways that were affected by heat stress. Overall, these results show distinct cellular responses to heat stress in two different genetic groups of cattle. This also highlight the long-term adaptation of *B. indicus* (Hariana) to tropical climate as compared to the crossbred (Vrindavani) with mixed genetic makeup (*B. indicus* X *B. taurus*).

**Supplementary Information:**

The online version contains supplementary material available at 10.1186/s41021-023-00271-8.

## Introduction

Dairy farming significantly contributes to the socio-economic development of communities, countries and regions [1, 2]. Dairy milk has nutritional value. Consequently, there is an increasing demand for milk and milk products in the human diet [3, 4]. Bovine milk is a primary source (up to 83%) of all the total diary milk production throughout the world [5]. Among several challenges faced by the cattle farming, environmental stress is considered as one of the major threats for sustainability of higher milk production [6].

Impending climate change scenario has remained as an unanimously accepted reality [7]. The Intergovernmental Panel on Climate Change (IPCC) has predicted temperature rise of 1.5 °C in the next two decades [8]. The climate change has a complex effect on the livestock production. Temperature stress would be one major environmental factor to influence the health and productivity of dairy cattle [9]. For example, when the outside temperature reaches to 35 °C, quantity of milk is decreased by 33% and at the temperature of 40^o^C by 50% [10]. Heat stressed cows at higher temperature humidity index (THI = 83.4) have had significantly lesser milk yield (31.2 vs. 38.6 kg/d) than the control cows at thermo-neutral conditions (THI = 75.7) [11]. Similar effects on the milk yield were observed during the season with high temperature and humidity [12, 13]. These deleterious effects on milk production and health of animals stem from the heat stress induced molecular changes that alter normal physiological mechanisms [14, 15].

Gene networks within the cells and tissues co-ordinate metabolism and milk production in dairy cattle [16]. The most recognized pathways of thermal stress responses include the central role of heat shock proteins (*HSP*) [17]. Heat shock proteins are associated with heat stress response in cattle [18]. Apart from heat shock proteins, recent genome-wide expression studies identified differences in the other transcripts between heat stressed and their respective control cells (bovine mammary epithelial cells and peripheral blood mononuclear cells) [19]. An iTRAQ proteomic study revealed heat stress induced changes in mammary tissues of dairy cows, independent of feed intake by the animals [20]. Relatively heat tolerant and susceptible cattle respond differently to heat stress. The comparative study on relatively heat tolerant and susceptible dairy cattle identified potential regulatory genes responsible for differences in heat stress response mechanisms [21]. Similar approach with limited data found gene expression differences between the zebu and crossbred cattle [22, 23].

The tropical Zebu cattle (*B. indicus*) are low producers compared to their European counterparts (*B. taurus*). However, *B. indicus* are relatively tolerant to environmental heat stress owing to their long-term adaptation in the tropical climate conditions [24]. Cross-breeding programs were initiated between *B. indicus* and exotic breeds (Holstein Friesian, Jersey and Brown-Swiss) with the aim to enhance the milk production [25–27]. In the Indian subcontinent, this has resulted in new breeds of dairy cattle (Jersind, Jerthar, Karan-Swiss, Karan fries, Sunandini, Frieswal, Phule Triveni and Vrindavani) and these synthetic animals with exotic genetic background are capable of producing more milk than the native Indian breeds [28]. However, these animals are relatively susceptible to environmental stress as they are yet to be adapted to tropical climatic conditions [29]. The susceptibility of the crossbred dairy cattle to environmental stress is considered as a major drawback of the cross breeding program. In the recent years, environmental extremes caused by global warming puts them at higher risk of being affected by heat stress. Enhancement of thermo-tolerance (with no adverse effect on milk yield) in crossbred cattle would make them the most desirable population for the tropical dairy.

Any intervention to enhance the thermo-tolerance in crossbred animals would require thorough understanding of molecular mechanism regulating thermo-tolerance. Towards this end, differences in heat stress responses and underlying molecular changes between the zebu and crossbred cattle need to be delineated. In the present study, we compared heat stress response between Hariana breed (*B. indicus*) and its crossbred counterpart, Vrindavani (Holstein–Friesian/ Brown Swiss /Jersey [50–75%] X Hariana [25–50%]). Our hypothesis included: 1) heat stress dysregulates multiple genes as a part of intricate network of cellular stress response; 2) genetic background of cattle (*B. indicus* versus *B. indicus* X *B. taurus*) contributes to differences in cellular stress responses. Our genome-wide expression analysis revealed activation of distinct genes during heat stress with notable differences between Hariana and Vrindavani cattle. The functional analysis revealed potential molecular mechanisms involved in cellular heat stress responses in these animals.

## Materials and methods

### Experimental animals and design

Animals from Indian native Zebu (Hariana) and its corresponding crossbred, Vrindavani (Hariana [25–50%] X Holstein–Friesian/ Brown Swiss /Jersey [50–75%]) were included in the present study. Six healthy male animals (12–18 months and *N* = 3 per breed) housed at ambient conditions with adequate feed and water. The experiments were approved by the Institute Animal Ethics Committee (IAEC) and conducted at Indian Veterinary Research Institute (IVRI), Bareilly, UP, India. The experiments were carried out during winter months (December to February) when environmental temperature ranges between 15–18 °C with 40–50% average humidity.

### PBMC isolation and in vitro heat shock treatment

Isolation of PBMC from blood samples of experimental animals and heat shock treatment were done as per the procedures explained in the earlier studies [30]. Briefly, the blood samples were collected from experimental animals in the morning hours (10.00 am) using sodium heparin coated vacutainers. Blood samples were diluted with PBS and layered on Histopaque-1077 (Sigma-Aldrich). Density gradient centrifugation was used for separation of peripheral blood mononuclear cells (PBMC). The isolated PBMCs were suspended in RPMI 1640 medium (Gibco, Thermo Scientific Inc.) supplemented with 10%FBS (Gibco) and antibiotic antimycotic solution (Gibco). PBMC were plated at 1 × 10^6^cells/well in 12-well plates and incubated at 37ºC in a humidified CO_2_ incubator (New Brunswick, Eppendorf SE). After 24 h of incubation, the control group cultures (CN) were exposed to 37 °C continuously, whereas the heat shock group (HS) cells were subjected to 42 °C for 3 h. After heat stress treatment, both CN and HS treated cells were processed for total RNA extraction.

### RNA isolation, cDNA library construction and sequencing

Total RNA were isolated from samples using Trizol (Ambion, Austin, TX, USA) as per the manufacturer’s protocol. RNA concentration, purity and integrity were first measured with NanoDrop ND-1000 UV–vis Spectrophotometer (NanoDrop Technologies) followed by RNA Nano 6000 Assay kit on Bioanalyzer 2100 system (Agilent Technologies). cDNA libraries were generated from total RNA with Illumina TruSeq RNA Sample Preparation v2 Kit (Illumina, San Diego, CA, USA) following manufacturer's instructions. Quality of sequencing libraries was assessed with Bioanalyzer 2100 system. Sequencing was performed for total 12 sequencing libraries using Illumina HiSeq 2500 and 100 bp paired sequencing reads were produced.

### Quality control and alignment to reference genome

Quality of the raw reads was assessed using FastQC. Adapters and low quality bases were trimmed with fastX clipper. The quality trimmed reads were separately aligned to the *B. taurus* genome (ARS-UCD1.2, GCA_002263795.2) using DRAGEN RNA splice aware aligner. Using annotation files (GTF), gene level and transcript level read count files were generated for all the samples for further analysis.

### Identification of differentially expressed genes (DEG)

Differentially expressed genes (DEG) were identified using the DESeq2 protocol. Transcripts per million (TPM) counts were subjected to shrinkage estimators for dispersions followed by fold change analysis with generalized linear model (GLM). Benjamini–Hochberg method was applied for calculation of false discovery rate (FDR) (Padj) values. The criteria of log_2_ fold change ≥ 1 with padj < 0.05 were used for selecting the DEG.

### Functional enrichment analysis and gene annotation

Gene Ontology (GO) and Kyoto Encyclopedia of Genes and Genomes (KEGG) databases were used for functional enrichment analysis. The analysis were performed based on DEGs identified between control (CN) and heat stress (HS) conditions for each breed of cattle. GO terms significantly associated with the gene lists were determined based on their FDR. Functional evidence of the relationship between the significant GO terms (FDR < 0.05) and the DEGs were identified. The Protein Analysis Through Evolutionary Relationships (PANTHER) [31] and Database for Annotation, Visualization, and Integrated Discovery (DAVID) 6.8 were used to perform functional annotation for the genes.

### Quantitative real-time PCR (qRT-PCR) validation

The total RNA was isolated from PBMC samples using TRIzol reagent (15,596,026, Thermo Scientific Inc.) following manufacturer's protocol. The purity was checked by nanodrop spectrophotometer (NanoDrop 3300, Thermo Scientific Inc). The total RNA was used for synthesis of cDNA using First strand cDNA synthesis kit (K1622 Revert Aid, Thermo Scientific Inc.). The gene expression changes were analyzed by using Brilliant III SYBR green kit (Agilent Technologies Inc.). Beta-Actin (*ACTB*) was used as internal control gene. Initial fold changes were calculated using 2^(–ΔΔCT) method later transformed into log_2 _fold change values. The list of primers designed and used in the gene expression studies is included as supplementary table (Table S1).

## Results

### Quality filtered RNA sequencing reads aligned to the bovine reference genome

A little more than 2.7 billion, 100-base paired-end total reads were generated in the present study. On an average there were 220 million reads per sample. More than 98% reads were mapped to the *B. taurus* reference genome (ARS-UCD1.2, GCA_002263795.2). The samples had an average of 18,493 genes detected that accounted for about 84.5% transcripts of all the 21,880 annotated cow genes (ARS-UCD1.2). The count data for each samples were filtered by removing the genes that had TPM smaller than 1 to eliminate genes with low counts. This resulted in final count table of 11526 and 11,174 genes respectively for Hariana (control and HS treated) and Vrindavani (control and HS treated) samples for differential expression of genes analysis. A summary of sequencing data, quality analysis and alignment results are included in Table 1. Exploratory analysis of the sample data revealed hierarchical clustering of control (CN) and heat stress (HS) samples in both Hariana and Vrindavani (Supplementary Fig. S1A and B). The principal component analysis (PCA) results show that the samples are grouped based on the heat stress treatment in both breeds indicating the effect of heat stress treatment on the overall gene expression patterns (Supplementary Fig. S1C and D).

**Table 1:** Overview of sequence data for control and heat stress group samples for Hariana and Vrindavani cattle and results of alignment to annotated reference of cattle genome

Sample id	Sample Name	Total reads	Total mapped reads	Unmapped reads	Mapping (%)	genes with read counts	Transcriptome (%)
H1CN	Hariana control1	237916210	235786737	2129473	99.11	18271	83.50
H2CN	Hariana control2	224288520	222119147	2169373	99.03	18144	82.92
H3CN	Hariana control3	239439060	236752849	2686211	98.88	18495	84.52
H1HS	Hariana heat stress1	229920202	227622020	2298182	99.00	18583	84.93
H2HS	Hariana heat stress2	239595592	237360061	2235531	99.06	18537	84.72
H3HS	Hariana heat stress3	216899590	214573568	2326022	98.93	18440	84.27
V1CN	Vrindavani control1	217530560	215501957	2028603	99.07	17932	81.95
V2CN	Vrindavani control2	216324790	213623203	2701587	98.75	18282	83.55
V3CN	Vrindavani control3	223854906	221561138	2293768	98.98	18196	83.16
V1HS	Vrindavani heat stress1	222051964	219925107	2126857	99.05	18605	85.03
V2HS	Vrindavani heat stress2	242652446	239450639	3201807	98.68	19627	89.70
V3HS	Vrindavani heat stress3	256578848	254061408	2517440	99.02	18812	85.97

### Heat shock alters the gene expression patterns in bovine PBMC

**Fig. 1:**
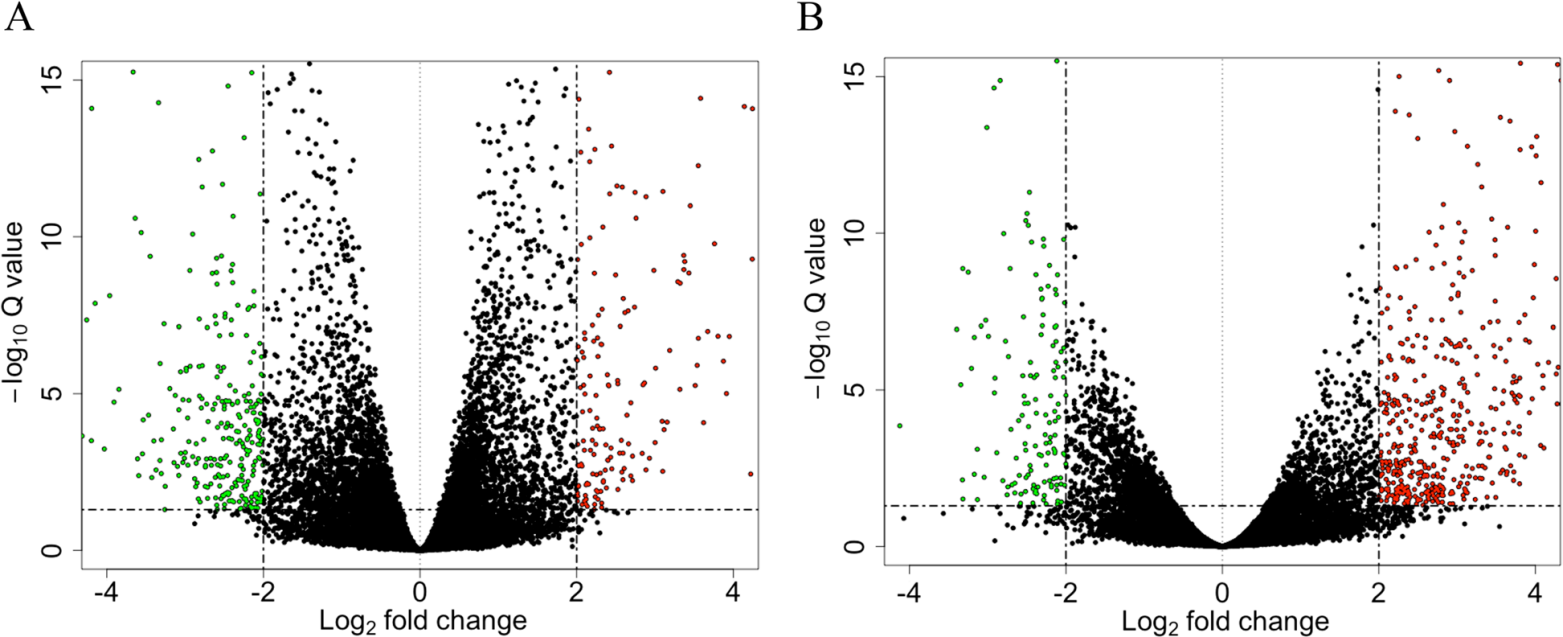
Representation of heat shock induced differential expression of genes in Hariana (**A**) and Vrindavani (**B**). The up-regulated genes were represented as red dots whereas the down-regulated genes were represented as green dots. The criteria of significance considered log_2 _fold change values higher than 2 with FDR value smaller than 0.05

**Fig. 2:**
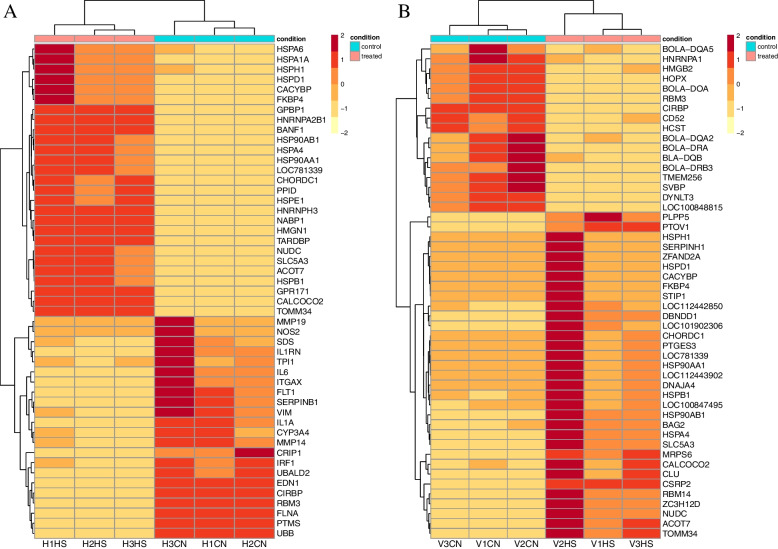
The pattern of heat shock induced gene expression in Hariana (**A**) and Vrindavani (**B**). The differentially expressed genes were represented based on the normalised gene counts across all the three (*N* = 3) replicates for each breed of cattle. The hierarchical clustering of the replicate samples clusters together based on the heat stress treatment

Differential expression analysis between the control (CN) and heat stress (HS) treatment group (FDR < 0.05 and log_2 _fold change > ± 1.0) revealed multiple DEG (differentially expressed genes) in both Hariana and its crossbred, Vrindavani. These results are represented through volcano plot (Fig. 1A and B). In Hariana breed, there were 368 DEG, of which 130 were up-regulated and 238 were down-regulated. Similarly, in Vrindavani breed, there were 337 DEG, of which 139 were up-regulated and 198 were down-regulated. The major DEG depicted in the heat map showing their fold change of expression based on the normalized counts of all individual samples (*n* = 3) of each control and heat stress group (Fig. 2A and B). Based on the lowest FDR and highest log_2 _fold change the top 10 up-regulated and top 10 down-regulated genes are presented in the Table 2.

**Table 2:** The list of top 10 up-regulated and 10 down-regulated genes in *B. indicus* (Hariana) and its cross-breed of *B. taurus* (Vrindavani) cattle

**Genes**	**log2FoldChange**	**padj(FDR)**
***TOP 10 Up-regulated genes in Hariana breed***		
*HSPB1*	3.97	1.34E-49
*SLC5A3*	3.49	1.70E-08
*HSPA1A*	3.14	8.04E-11
*FKBP4*	2.84	5.49E-17
*LOC781339*	2.70	7.91E-17
*HSP90AA1*	2.70	7.41E-25
*HSPA6*	2.56	5.91E-05
*HSPA4*	2.46	1.57E-17
*ACOT7*	2.37	3.94E-09
*HSPH1*	2.23	3.02E-05
***TOP 10 Down-regulated genes in Hariana breed***		
*NOS2*	-5.12	1.77E-12
*MMP19*	-4.25	8.91E-06
*IL1RN*	-3.62	9.58E-14
*SDS*	-3.52	6.39E-09
*IL6*	-2.88	2.24E-08
*EDN1*	-2.69	9.91E-10
*ITGAX*	-2.63	4.66E-06
*CYP3A4*	-2.44	1.98E-06
*FLT1*	-2.39	1.20E-05
*TPI1*	-2.37	2.67E-05
***TOP 10 Up-regulated genes in Vrindavani breed***		
*BAG3*	5.65	0.000188988
*FKBP4*	5.53	7.86E-15
*ZFAND2A*	5.51	1.03E-12
*SLC5A3*	5.35	7.86E-15
*HSPB1*	5.24	3.26E-22
*DNAJA4*	4.96	1.70E-10
*HSP90AA1*	4.40	8.37E-16
*LOC112442850*	4.11	3.32E-05
*CLU*	3.86	1.98E-07
BAG2	3.79	1.89E-08
***TOP 10 Down-regulated genes in Vrindavani breed***		
*RBM3*	-2.94	2.88E-20
*BOLA-DRA*	-2.62	8.63E-09
*BOLA-DQA5*	-2.45	5.24E-05
*HCST*	-2.39	4.89E-07
*SVBP*	-2.36	7.74E-05
*BOLA-DQA2*	-2.30	4.11E-05
*IDO1*	-2.29	0.000125643
*HOPX*	-2.19	1.32E-07
*BOLA-DOA*	-2.18	4.11E-05
*LOC100848815*	-2.12	6.72E-07

### Zebu and crossbred cattle show differences in heat induced gene expression

**Fig. 3:**
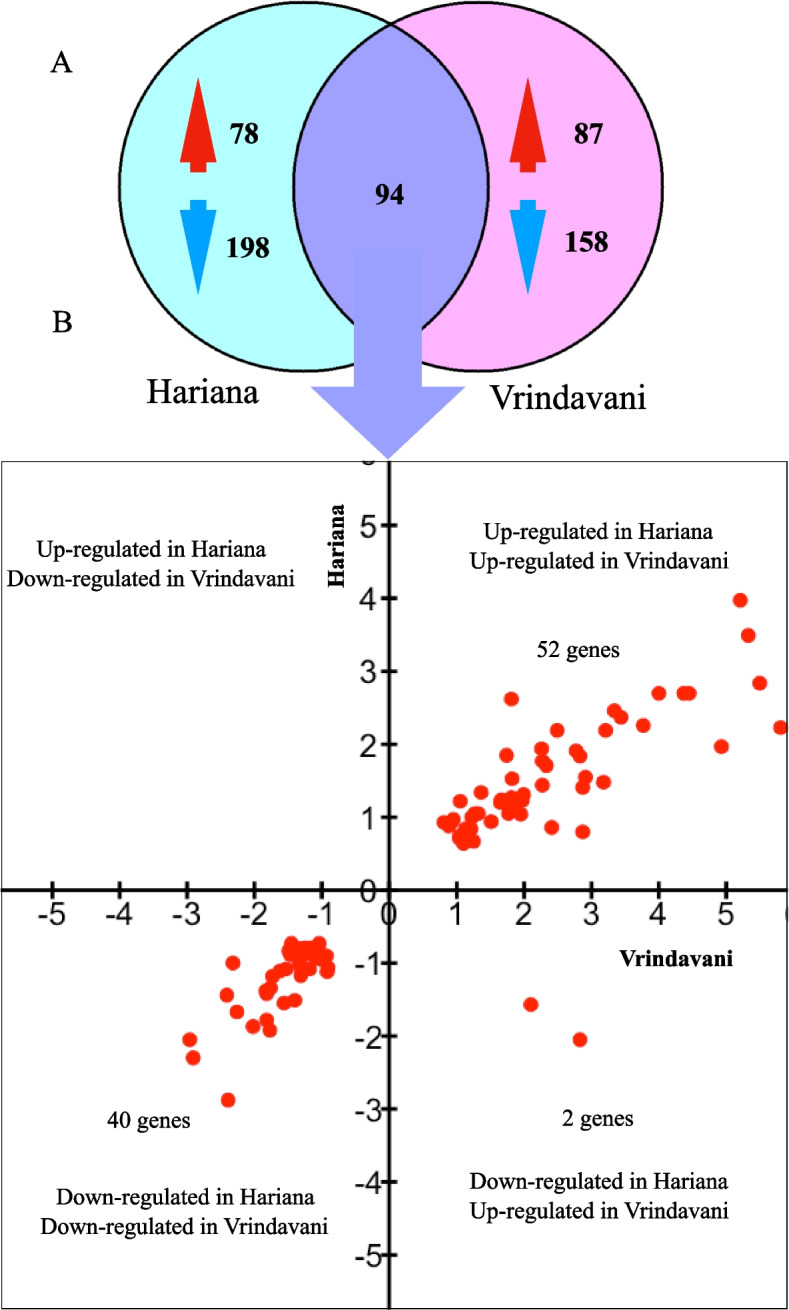
Differences in heat shock induced gene expression in Hariana and Vrindavani represented through Venn diagram (**A**). The common DEG that were shared were further categorized in quadrant plot showing their different possible expression (**B**)

After identifying the heat stress induced gene expression changes separately in each breed of cattle, we set out to find the differences between these breeds. Out of 368 and 337 DEG identified in response to heat stress treatment in Hariana and Vrindavani respectively, 94 genes were common between both the breeds (Fig. 3). Among these 52 common DEG were up-regulated, while 40 common DEG were down-regulated. However, there were two genes (*PPP1R15A* and *HMOX1*) that were up-regulated in Vrindavani but down-regulated in Hariana. Apart from these common DEG, 276 and 245 distinct DEGs were unique to for Hariana and Vrindavani cattle, respectively.

### Differentially expressed genes are related to significant cellular functions

**Fig. 4:**
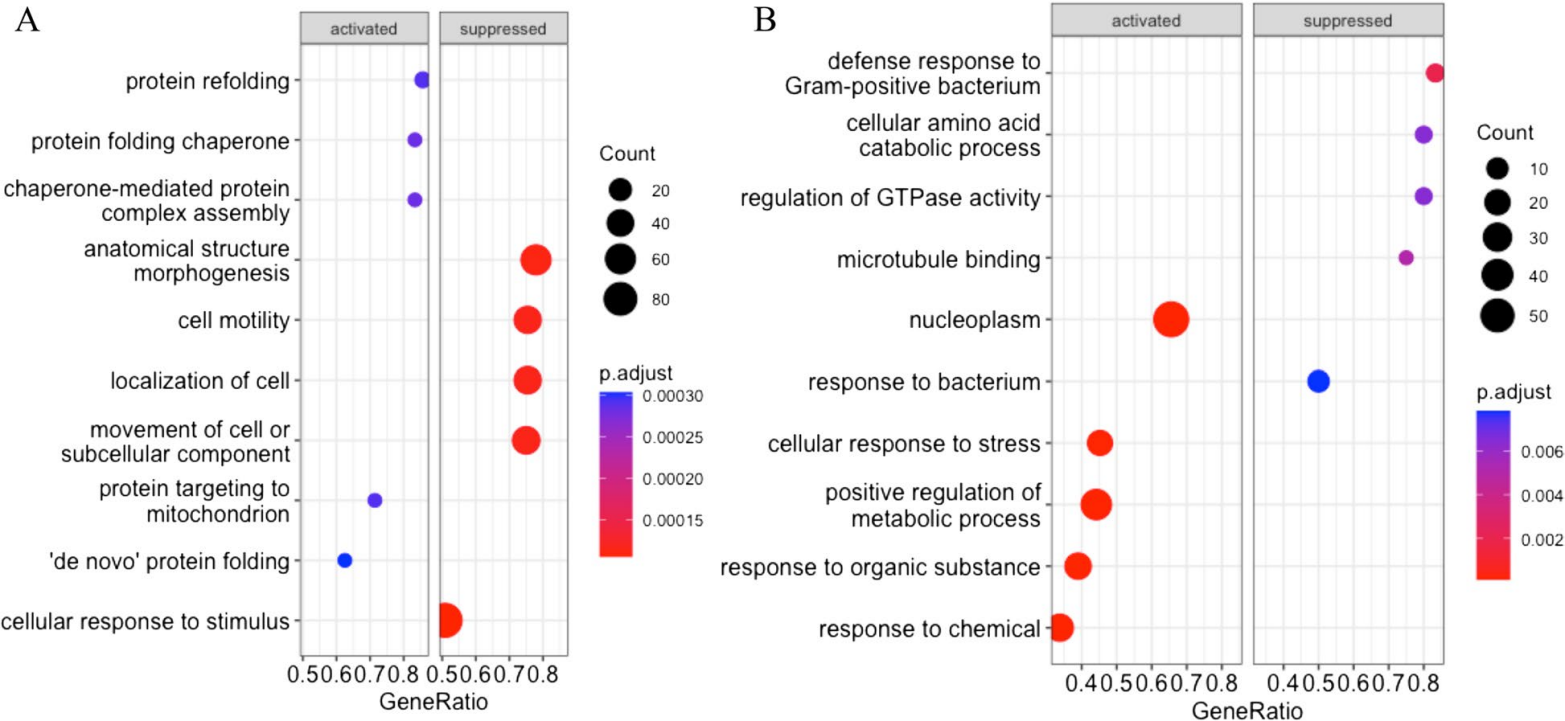
Functional annotation of DEG under heat stress. Gene enrichment analysis for Gene ontology (GO) terms and top 5 activated and top 5 suppressed terms were represented for Hariana (**A**) and Vrindavani (**B**). The size of the dot indicates number of genes enriched and color indicates the statistical significance (FDR value)

**Fig. 5:**
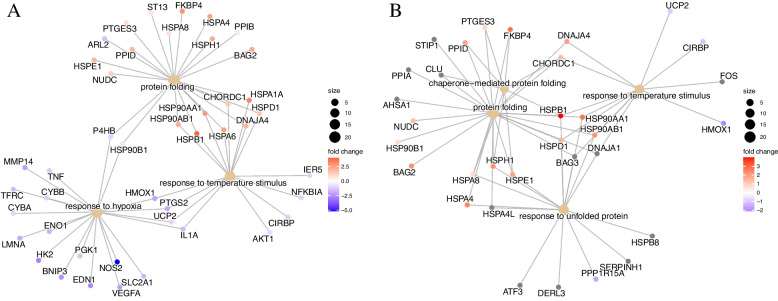
Genes connecting to the major biological process obtained from gene set enrichment analysis for Hariana (**A**) and Vrindavani (**B**). Size of the biological process indicates the number of genes connecting to it and the color of genes show their expression in terms of fold change

To determine biological functions of the heat shock induced genes at molecular level, the DEG were subjected to the gene set enrichment analysis with GO and KEGG databases. The enriched GO terms were categorized as activated or suppressed. In case of Hariana breed heat stress treatment activated mainly chaperone mediated protein folding and refolding. The suppressed biological processes included cell motility, localization of cells and cellular response to stimulus (Fig. 4A). In Vrindavani, the heat stress treatment activated cellular response to stress, positive regulation of metabolic process and responses to inorganic substance or chemicals. The suppressed biological processes include possible defense response to Gram-positive bacterium, cellular amino acid catabolic process, regulation of GTPase activity and microtubule binding (Fig. 4B). The majority of the genes involved in activation of protein folding, refolding and response to temperature stimulus had higher expression whereas the majority of the genes involved in response to hypoxia had lower expression in both Hariana and Vrindavani cattle (Fig. 5A and B).

**Fig. 6:**
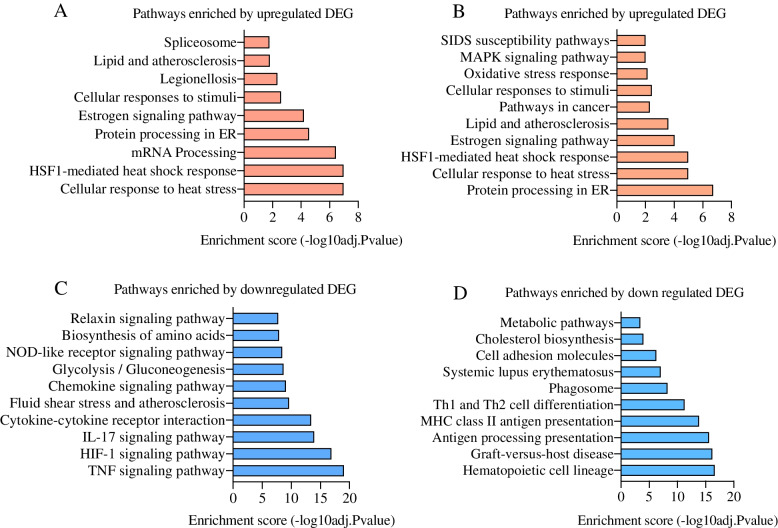
Signaling pathways affected by the DEG obtained from heat stress treatment. The pathways enriched by the up-regulated genes in Hariana (**A**) and Vrindavani (**B**). Similarly, the pathways enriched by the down-regulated genes in Hariana (**C**) and Vrindavani (**D**) were presented

Further, the differentially expressed genes (DEG) were used to know the enrichment of different cellular signaling pathways. In Hariana cattle, the 5 major pathways enriched by upregulated DEG included cellular response to heat stress, regulation of *HSF-1* mediated heat shock response, mRNA processing, protein processing in endoplasmic reticulum and estrogen signaling pathway (Fig. 6A). The activated pathways in Vrindavani cattle are similar to those of Hariana cattle, except that the lipid and atherosclerosis pathway was enriched instead of the mRNA processing pathway (Fig. 6B). The potentially suppressed pathways in Hariana included *TNF* signaling pathway, *HIF-1* signaling pathway, *IL-17* signaling pathway, cytokine-cytokine receptor interaction and fluid shear stress and atherosclerosis (Fig. 6C). However, differences were observed in Vrindavani in terms of potentially suppressed pathways such as hematopoietic cell lineage, graft-versus-host disease, antigen processing presentation, MHC class II antigen presentation and Th1 and Th2 cell differentiations (Fig. 6D).

### Major categories of genes are affected by heat stress in zebu and crossbred cattle

**Fig. 7:**
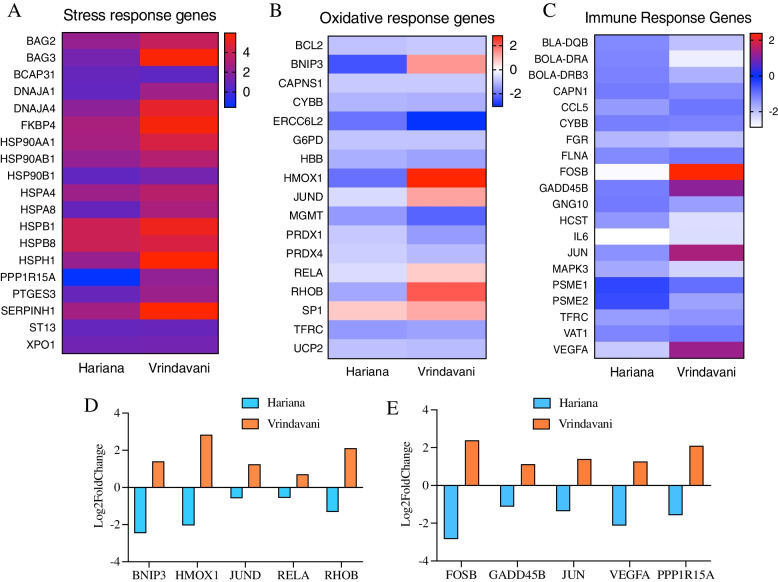
Comparison of expression patterns of different categories of genes between Hariana and Vrindavani. The heat map shows expression differences of heat stress response genes (**A**), oxidative stress response genes (**B**) and immune response genes (**C**). The bar diagram shows significantly different expression patterns of genes involved in oxidative stress response (**D**) and immune response (**E**)

To narrow down the differences between breeds, DEG obtained after analysis were categorized based on their biological functions. Three major categories such as stress response genes, oxidative response genes and immune response genes were compared for their expression patterns between the breeds (Fig. 7). The heat stress response genes in the two cattle breeds displayed different patterns of gene expression, but these differences were merely in the degree of gene expression, as all genes in this category were upregulated in both breeds. However, crossbred cattle (Vrindavani) showed higher degree of up-regulation for most of the genes compared to the native zebu cattle (Hariana) (Fig. 7A). In case of the oxidative response category, the genes such as *BNIP3*, *HMOX1*, *JUND*, *RELA* and *RHOB* were found up-regulated in the Vrindavani cattle, whereas the same were down-regulated in the Hariana cattle (Fig. 7B and D). Similarly, in the immune response category, the major genes such as *FSOB*, *GADD45B*, *JUN*, *VEGFA* and *PPP1R15A *were up-regulated in Vrindavani, whereas the same were down-regulated in the Hariana cattle (Fig. 7C and E). These results indicated that Vrindavani and Hariana cattle show differences in the expression patterns of certain immune response and oxidative response genes when challenged with the heat stress.

### Protein–Protein interaction networks indicated heat shock response

**Fig. 8:**
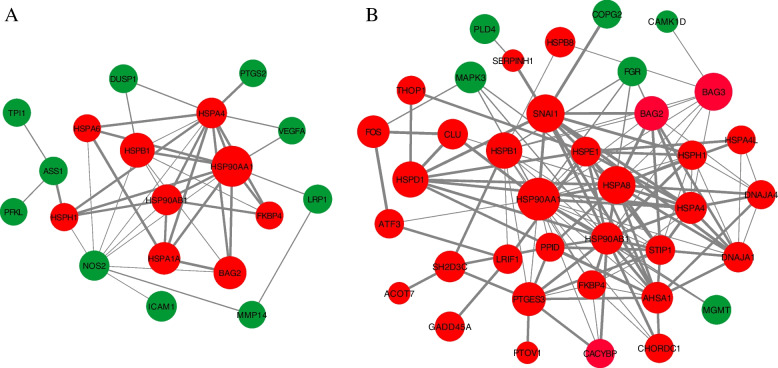
Protein–protein interaction (PPI) network of hub genes for Hariana (**A**) and Vrindavani (**B**). The size of edges indicate the degree of connections of the respective genes and width of node lines indicate the strength of interactions. Up-regulated genes are marked red and down-regulated genes are marked green

The protein–protein interaction networks were constructed by the involvement of differentially expressed proteins. The BioGRID protein interaction network repository was used for extracting the interactions. Further, differentially expressed highly connected genes (DEHC) (fold change >  ± 2 and padj < 0.05 with degree of connections > 20) were used to determine potential and significant protein–protein interaction networks (Fig. 8A and B). There were 23 and 49 DEHC genes in Hariana and Vrindavani cattle, respectively. Out of DEHC genes, 17 were found common between Hariana and Vrindavani cattle. Most of these proteins represented major hub proteins such as *HSP90AA1*, *HSPB1*, *HSPD1*, *CALCOCO2*, *BAG2*, *HSP90AB1*, *HSPA4*, *MAPK3*, *ACOT7* and *HSPE1*.These genes were related to upregulated genes of heat shock, oxidative stress and immune related genes. Among these genes, only *MAPK3* was found down-regulated and the remaining genes were up-regulated. The change in expression of major hub proteins indicates their potential importance in coordinating the cellular heat stress response.

### Validation of RNA sequencing results using qRT-PCR

**Fig. 9:**
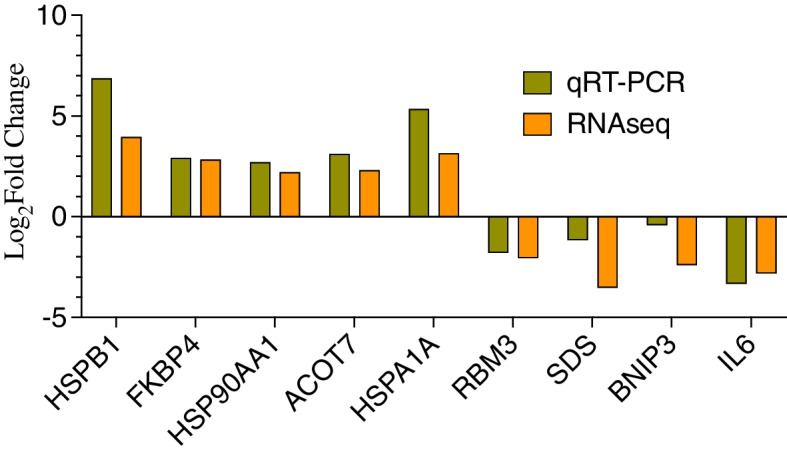
Validation of major differentially expressed genes using qRT-PCR. The up-regulated genes (*HSPB1*, *FKB4*, *HSP90AA1*, *ACOT7*, *HSPA1A*) and down-regulated genes (*RBM3*, *SDS*, *BNIP3*, *IL6*) as identified in RNA sequencing data were evaluated using qRT-PCR. The results of qRT-PCR were compared with the expression levels (log_2 _fold change) obtained in the RNA sequencing

Five major up-regulated genes (*HSPB1*, *FKBP4*, *HSP90AA1*, *ACOT7*, *HSPA1A*) and four down-regulated genes (*RBM3*, *SDS*, *BNIP3* and *IL6*) as observed in the RNA sequencing results were analyzed with qRT-PCR. The expression of these genes obtained through qRT-PCR is presented in comparison with the results of RNA sequencing (Fig. 9). The direction and the level of expression (Log_2_fold change) of these genes indicated that qRT-PCR and RNA sequencing results are similar and comparable.

## Discussion

Heat stress adversely impacts health, production and reproduction parameters in dairy cattle [32–34]. Curtailing the effects of heat stress and its consequent losses has been an important objective of farmers and animal researchers. One of the strategies is to understand the heat stress responses and utilize the better adaptability of animals to reduce the impact of heat stress [35, 36]. The present study delineates the molecular mechanisms of heat stress response in cattle of different genetic background. We compared genome-wide expression patterns between relatively thermo-tolerant breed of *B. indicus* (Hariana) and its crossbred, *B. indicus X B. taurus* (Vrindavani).

Different types of heat shock proteins (*HSP90*, *HSP70*) and other related candidate genes have been individually characterized in zebu and crossbred cattle [37, 38]. However, stress response is a complex biological process regulated by multiple genes [39]. Transcriptomics using high-throughput sequencing and other techniques allow capture of genome-wide expression patterns [40–42]. Using this approach, the present study identifies the involvement of multiple genes that act through interacting networks during cellular stress response. There were 368 and 337 DEG in the heat stress exposed Hariana and Vrindavani cattle respectively. The up-regulated genes mainly belong to the molecular chaperones (*HSPB1*, *HSPA1A*, *HSP90AA1*, *DNAJB1*), co-chaperone gene (*FKBP4*) and solute carrier family (Sodium/Myo-Inositol Cotransporter) protein (*SLC5A3*). The major down-regulated genes were immune response genes such as interluekin-6 (*IL6*), interleukin 1 receptor antagonist (*IL1RN*), Nitric oxide synthase 2 (*NOS2*) and bovine major histocompatibility complex (*BoLA*) genes that play important role in antigen presentation. Earlier, similar transcriptomics approach identified DEG in response to heat stress in cattle [43]. Heat stress induced up-regulation of heat shock genes (*DNAJB1*, *HSPA1A*, *HSP90AA1*, *HSPB8*, *DNAJB4*) was similarly found in PBMC of Holstein dairy cattle [39]. Apart from up-regulated heat shock genes, interestingly, we found expression of immune response related genes down-regulated. These findings were also corroborated by earlier studies that showed down-regulated expression of *BoLA* (*BoLA-DRB3*) in bovine mammary cells [44]. The down-regulation of major immune response genes indicates that animals become more susceptible to infection during the heat stress period.

Many of the genes identified in the present study were found related the heat tolerance traits of cattle in the previous studies [45–47]. Polymorphisms in different heat shock proteins (*HSP90*, *HSPA6, HSPB7*) and heat shock factor 1 (*HSF1*) were associated with the heat tolerance traits in cattle [45, 48, 49]). In addition, mutations in other related genes such as prolactin (*PRL*) and prolactin receptor (*PRLR*) were associated with thermoregulation and hair morphology in cattle [50].

The present study recorded GO enrichment for DEGs as activation of cellular response to stress and chaperone mediated protein folding. In contrast to Hariana (zebu) cattle, heat stress in Vrindavani (crossbred) cattle resulted in a suppressed response to bacteria and defense response to Gram-positive bacteria, suggesting that the Vrindavani cattle is more susceptible to bacterial infection during heat stress period. Experiments in Holstein calves revealed that heat stress induced genes were mainly categorized for protein folding functions [51]. PBMCs of Holstein dairy cattle subjected to heat stress showed enrichment of similar biological process such as protein folding, response to endoplasmic reticulum stress, negative regulation of cell proliferation, inclusion body assembly and apoptotic process [39]. We found major enriched signaling pathways by up-regulated DEGs such as HSF1mediated heat shock response, estrogen signaling pathway, *MAPK* singling pathway. Whereas the down-regulated genes enriched mainly immune responses such as *TNF* signaling, *IL-17* signaling, Chemokine signaling and MHC class II antigen presentation. These results indicate that heat stress activates protective mechanisms to prevent damage or misfolding of cellular proteins. However, major immune response pathways were dampened indicating the vulnerability of animals to possible challenge of infectious agents. In earlier studies, heat stress to Jersey and Holstein steers was found to alter the proportion of immune cells and expression of cytokines (*IL-10*, *IL-17A* and *IL6*) indicating enhanced susceptibility of heat-stressed steers to diseases [52]. The effect of heat stress on immune cells and gene expression pattern was also found breed specific for Holstein and Jersey cattle [52, 53]. Conversely, different immune categories of Holstein cows (ranked as high, average and low immune responders) were found to exhibit differences in the heat stress response [54]. One of the highlight of our study is that there are differences in the expression of immune system related genes between Hariana and Vrindavani in responses to heat stress. The immune pathways of Vrindavani cattle were found to be more dysregulated in comparison to Hariana cattle when subjected to heat stress. These results may suggest that Vrindavani cattle are more vulnerable to infections during heat stress compared to Hariana cattle.

Our analysis focused on comparison of DEG and it revealed up-regulation of certain oxidative stress related genes (*BNIP3*, *HMOX1*, *VEGFA*, *RHOB*) in Vrindavani cattle. However, those genes were down-regulated in Hariana cattle. Bcl2 interacting protein 3 (*BINP3*) was found induced in hypoxic stress [55]. BNIP3 was found to promote apoptosis in response to energy stress by inhibiting mTORC1 function [56]. Heme oxygenase-1 (*HMOX1*) was reported to be upregulated during different types of stressors mainly during cellular oxidative stress response [57, 58]). Role of *HMOX1* was highlighted in protecting the cells [59, 60]. The distribution of *HMOX1*, *HSP70* and glutathione together depict cellular stress tolerance [61]. Vascular endothelial growth factor-A (*VEGFA*) is a major hypoxia response protein induced by hypoxia inducible factor alpha (*HIF1α).* However, it is also upregulated without the help of *HIF1α* during unfolded protein response (UPR) [62]. Heat stress treatment in rats significantly increased expression of *VEGF* along with *HSP70* [63]. *VEGF* expression was up-regulated in heat stressed Jersey cattle. *VEGF* signaling pathway plays an important role in immunosuppression and oxidative stress response in the affected animals [43]. Small GTP binding protein RhoB (*RHOB*) was found rapidly up-regulated by genotoxic and heat stress [64]. Upregulation of RhoB significantly inhibited heat stress induced apoptosis and played cytoprotective role [65].

Apart from differences in the oxidative stress response gene, certain immune response related (*FSOB*, *JUN*, *GADD45B*) genes were found up-regulated in the Vrindavani, whereas those genes were found down-regulated in the Hariana cattle. *FSOB* and *JUN* proteins are produced in response to any cellular stimuli and contributes to the formation of transcription factor activator protein-1 (*AP-1*). Activation of *AP-1 *in turn regulates genes involved in proliferation, migration and immune response [66–68]. Studies on rats indicated that FOSB proteins potentially have a role in long-lasting adaptation changes in the cellular stress responses [69]. In the present study, we found significant up-regulation of *FSOB* in Vrindavani indicating its possible role in differential heat stress response compared to the Hariana cattle. Also, Vrindavani cattle are likely to actively undergoing adaption process to tropical climate. Under environmental stressors, there was an up-regulation of *GADD45B* (growth arrest DNA damage inducible beta protein) gene expression and this causes arrests of cells growth or proliferation. *GADD45B* activates *p38/JNK* pathway via *MTK1/MEKK4* kinase [70]. Earlier it was shown that *GADD45B* levels were increased in stress susceptible but not resilient mice [71]. This finding corroborates with our results as relatively heat stress susceptible crossbred Vrindavani cattle showed higher expression of *GADD45B* compared to Hariana cattle. Further, interestingly its important paralogue *GADD45A* showed significantly differential methylation in Angus cattle during heat stress challenge [72]. Hence based on the long-term adaptation of animals to certain environmental conditions, epigenetic mechanisms may play a role in producing differences in stress responses.

## Conclusions

The present study revealed genome-wide expression patterns in the PBMC of cattle subjected to heat stress. Apart from heat stress response, we found mainly altered oxidative stress and immune response mechanisms as the genes related to these processes were found dysregulated. There were differences in the number and types of dysregulated genes categorized as common and specific for each of these breeds. Even though with same expression patterns, heat stress response genes (*HSPH1*, *HSPB8*, *FKB4*, *DNAJ4* and *SERPINH1*) were up-regulated at higher fold change in Vrindavani compared to the Hariana cattle. Further, certain oxidative stress response genes (*HMOX1*, *BNIP3*, *RHOB* and *VEGFA*) and immune response genes (*FSOB*, *GADD45B* and *JUN*) were up-regulated in Vrindavani whereas the same were down- regulated in Hariana cattle. These differences in cellular stress responses highlight the long-term adaptation of *B. indicus* (Hariana) to tropical climate compared to the mixed breed (Vrindavani) with contribution from *B. taurus.*

## Supplementary Information

**Additional file1: Fig. S1.** Exploratory analysis for genome-wide expression patterns of samples. **Table S1.** Primer sequences used in qRT-PCR for gene expression analysis. **Table S2.** Major heat shock induced differentially expressed genes and their functions.

## Data Availability

Data and materials presented in the manuscript will be made available upon request to the corresponding author.
