# Supplementary material for: Genome-wide expression analysis reveals different heat shock responses in indigenous (Bos indicus) and crossbred (Bos indicus X Bos taurus) cattle
Source: Genes Environ. 2023 May 1;45:17. doi: 10.1186/s41021-023-00271-8 (PMC10152620; doi:10.1186/s41021-023-00271-8)
Supplement: Supplementary file 1 — Additional file1: Fig. S1. Exploratory analysis for genome-wide expression patterns of samples. Table S1. Primer sequences used in qRT-PCR for gene expression analysis. Table S2. Major heat shock induced differentially expressed genes and their functions. [file 41021_2023_271_MOESM1_ESM.docx]

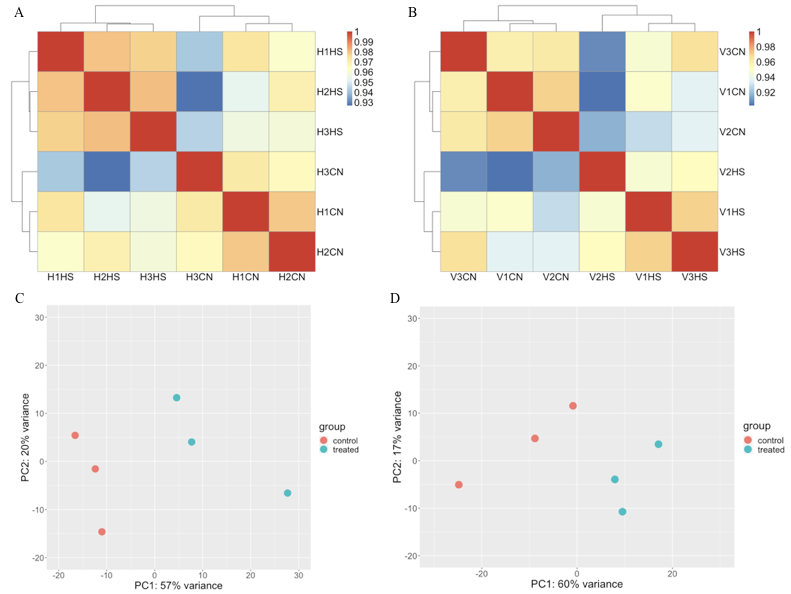


Fig. S1. Exploratory analysis for genome-wide expression patterns of samples. Correlation matrix and hierarchal clustering of samples show grouping of samples based on heat stress treatment in Hariana (A) and Vrindavani (B). The principal component analysis revealed segregation of samples for heat stress treatment in Hariana (C) and Vrindavani (D)

Table S1: Primer sequences used in qRT-PCR for gene expression analysis

| Gene name | Primer name | Primer sequence (5' to 3') |
| --- | --- | --- |
| HSPB1 | HSPB1_f | TGACGGTCAAGACCAAGGA |
|  | HSPB1_r | GAGTGAAGCAACGGGAAATGTA |
| FKBP4 | FKBP4_f | CTGGCCATGTGTCATCTGAA |
|  | FKBP4_r | GGAAGAGGCCTTTCTCATTGT |
| HSP90AA1 | HSP90AA1_f | TTGCCAAGTCTGGCACTAAA |
|  | HSP90AA1_r | CCACTAGGTAGGCAGAGTAGAA |
| ACOT7 | ACOT7_f | ATCACCTACACCTCCAAGCA |
|  | ACOT7_r | TACCACAGGGTGGCCTTATT |
| HSPA1A | HSPA1A_f | AGCCTGGAGAGAGCTGATAA |
|  | HSPA1A_r | ACAGGATCAACGACGTGAAG |
| RBM3 | RBM3_f | GCTTTGGACCTATTTCTGAGGT |
|  | RBM3_r | CGGATTGGTGAAGGTGATGAA |
| SDS | SDS_f | TGGTGGCGCTCAAGAAG |
|  | SDS_r | CCAGCAGACACAGAACACTAT |
| BNIP3 | BNIP3_f | GTGTTCCCGACTCTGTTTCTATT |
|  | BNIP3_r | GAAGCTCTGTTAGTGTCCTGTG |
| IL6 | IL6_f | AGACTACTTCTGACCACTCCA |
|  | IL6_r | GCTGCTTTCACACTCATCATTC |
| ACTB | ACTB_f | GCCTCCTATGTGGATGATGAAG |
|  | ACTB_r | GATCTCCATGTCATCCCAGTTG |

| **Name of the Gene** | **Important biological functions** |
| --- | --- |
| ***Key up-regulated genes in Hariana (B. indicus) and Vrindavani (B. indicus X B. taurus)*** | |
| HSPB1 (Heat shock protein family B member 1) | Involved in maintaining proteins in a folding-competent state. It plays a role in actin organization and stress resistance. |
| SLC5A3 (Solute carrier family 5 member 3) | Involved in cellular osmoregulation, potassium channel regulator function and inositol metabolic process. |
| HSPA1A (Heat shock protein family A member 1A) | Involved in preventing aggregation of proteins during cellular stress, degradation of misfolded proteins and protein quality control. |
| FKBP4 (FKBP prolyl isomerase 4) | Involved in immunoregulation by binding to the immunosuppressant elements, protein folding and their trafficking |
| HSP90AA1 ( Heat shock protein 90 alpha A member 1) | Involved in proper folding of target proteins by ATPase activity and its functions are modulated by other co-chaperone proteins. |
| DNAJA4 [DnaJ heat shock protein family (Hsp40) member A4] | Involved in binding to other chaperone proteins, inhibiting endothelial cell migration and inclusion body formation. |
| CPA5 (Carboxypeptidase A5) | Involved in folding of the active carboxypeptidase domain that involved in cleavage of other proteins to generate active peptides. |
| ACOT7 (Acyl-CoA thioesterase 7) | Involved in hydrolysis of the CoA thioester of palmitoyl-CoA to increase intracellular level of free fatty acids. |
| GAS8 (Growth arrest specific 8) | Involved in formation of the nexin-dynein regulatory complex (N-DRC), tumor suppressor activity and microtubule functions. |
| ***Key down-regulated genes in Hariana (B. indicus) and Vrindavani (B. indicus X B. taurus)*** | |
| NOS2 (Nitric oxide synthase 2) | Involved in formation of nitric oxide (NO) that mediates t bactericidal actions, inflammation and increases IL6 and IL8 |
| RBM3 (RNA binding motif protein 3) | Involved in cold shock response and low oxygen tension, enhances phosphorylation of translation initiation factors. |
| IL1RN (Interleukin 1 receptor antagonist) | Involved in inhibition of activities of interleukin 1 and modulates interleukin 1 related immune and inflammatory responses |
| SDS (Serine Dehydratase) | Involved in metabolizing serine & glycine amino acids and mediates protein homodimerization activity |
| IL6 (Interleukin-6) | Involved in inflammation, maturation of B cells, tissue regeneration, and metabolism. |
| EDN1 (Endothelin 1) | Involved in potent vasoconstriction, its cognate receptors are therapeutic targets for treatment of pulmonary arterial hypertension |
| ITGAX (Integrin subunit alpha X) | Involved in cell-cell interaction during inflammatory responses. It mediates monocyte adhesion and chemotaxis |
| CYP3A4 (Cytochrome P450 family 3 subfamily A4) | Involved in drug metabolism, synthesis of cholesterol, steroids and other lipids. |
| FLT1 (Fms related Receptor Tyrosine Kinase 1) | Involved in cell survival, cell migration, macrophage function and chemotaxis |
| BOLA-DRA (Bovine leukocyte antigen -major histocompatibility complex) | Involved in presenting peptides derived from extracellular proteins of pathogens to T cells and adaptive immune response |

Table S2: Major heat shock induced differentially expressed genes and their functions
